# Giant benign nodular hidradenoma of the shoulder: A rare tumor in orthopedic practice

**DOI:** 10.4103/0973-6042.79793

**Published:** 2010

**Authors:** Vibhore Singhal, Sansar C. Sharma, Juyal Anil, P. K. Sachan, Meena Harsh, Surina Singhal, Shailendra Raghuvanshi

**Affiliations:** Department of Orthopaedics, Himalayan Institute of Medical Sciences, Doiwala, Jolly Grant, Dehradun, Uttarakhand, India; 1 Department of Surgery, Himalayan Institute of Medical Sciences, Doiwala, Jolly Grant, Dehradun, Uttarakhand, India; 2 Department of Pathology, Himalayan Institute of Medical Sciences, Doiwala, Jolly Grant, Dehradun, Uttarakhand, India; 3 Department of Radiology, Himalayan Institute of Medical Sciences, Doiwala, Jolly Grant, Dehradun, Uttarakhand, India

**Keywords:** Axillary mass, nodular hidradenoma

## Abstract

A clear cell hidradenoma is a rare dermal tumor, which is believed to originate from the apical portion of the sweat glands. The usual size reported is 5–30 mm. It is generally found in the head, face, and upper extremity regions. This lesion has not been reported to be large enough to impinge a joint range of motion. Hence, its description in the orthopedic literature is extremely rare. We present a giant benign nodular hidradenoma presenting as painful restriction of the right shoulder joint in a 35-year-old male.

## INTRODUCTION

A nodular hidradenoma (NH) is a rare benign sweat gland tumor that is believed to originate from the eccrine gland.[1] The lesion is usually a single, slow-growing, well-marginated, round, mobile, cutaneous nodule ranging between 5 mm and 30 mm in diameter, and is rarely larger; lesions up to 6 cm have also been reported.[2] We encountered a tumor measuring 8 cm in the axilla of an adult male that restricted the shoulder range of motion, and remained a diagnostic dilemma till an excision biopsy was carried out and a histopathological report confirmed that it was a benign NH.

## CASE REPORT

A 35-year-old male presented with complaints of painful restriction of the right shoulder motion for the last 3 months. The pain was insidious in onset, dull-aching to pricking in character, occurring only while attempting extreme motions of the joint, more so in activities that involved raising the arm in an overhead position, e.g. bowling during the sport of cricket, reaching for articles placed high on the shelf or while sleeping on the affected side where it used to wake the patient in the night. There was no history of trauma, massage, morning stiffness, involvement of any other joint or any other medical or surgical history of illness. There was no history of any other lump in the body. On examination, the right shoulder appeared normal on inspection. There was no tenderness over the anterior, posterior or superior aspects of the shoulder as well as in the subacromion region. A mass was palpated in the right axilla, which was tender on deep palpation, well circumscribed, of the size of a small orange, firm in consistency and adherent to the underlying structures but not to the overlying skin. The mass became prominent on full abduction and used to move with the movement of the scapula. The mass was nonpulsatile and no bruit was heard on auscultation. The range of movement (ROM) was 0–160 degrees of abduction, with pain starting on 120 degrees and partially relieved on abduction beyond. Flexion, extension and internal rotation were comparable to the other (normal) side. There was 30 degrees of painful restriction of external rotation on the right side as compared with normal. The patient on further questioning revealed that this mass started as a small nodule observed while playing cricket 20 years back. The mass increased in size gradually but the patient ignored it till such time that he developed restriction of right shoulder movements, abnormal fullness and discomfort in the right axilla. Thorough clinical examination of the affected upper extremity was carried out to rule out any chronic infection, leading to lymphadenopathy. Examination of the other sites did not reveal any similar swelling or lymph node enlargement. There were no tingling sensation or numbness in the fingers or any wasting or hair loss noted in the right upper limb. Hematological tests were performed to rule out tumoral calcinosis (serum phosphate), complete hemogram, erythrocyte sedimentation rate and C-reactive protein for any possibility of occult systemic ailment manifesting as axillary mass.

**Figure 1:**
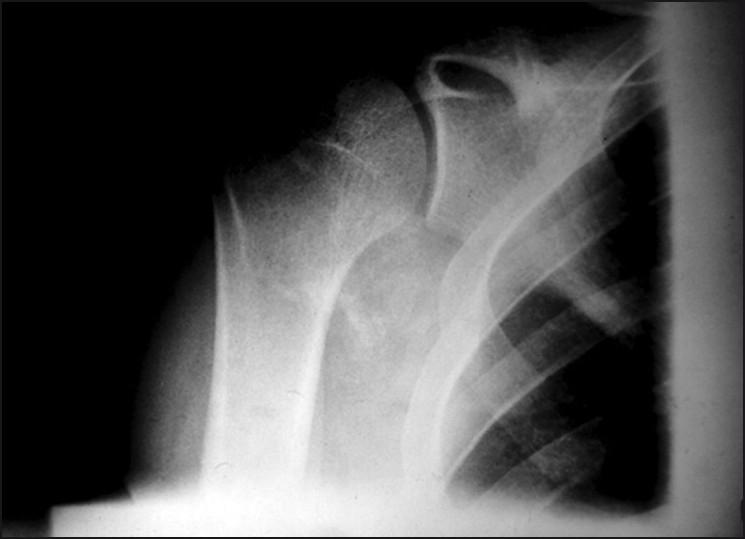
Preoperative photographs showing oval diffuse amorphous calcification in the axilla

A plain skiagram of the shoulder showed a round area of diffuse amorphous calcification in the axilla [Figure 1], whereas the shoulder joint was normal *per se*. The patient was suggested further investigation of the shoulder.

### Magnetic resonance imaging examination

**Figure 2:**
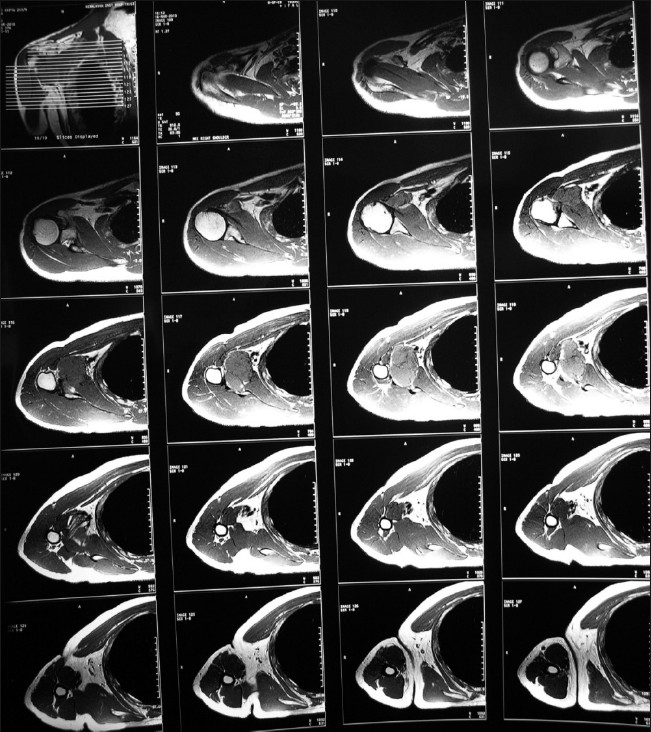
Magnetic resonance images showing the extent and location of the tumor. Note its relation with the subscapularis and the glenoid

A multiplaner magnetic resonance imaging (MRI) scan on a 1.5 Tesla magnet was performed and T1, T2 and proton density images were obtained using spin echo (SE) and gradient echo sequences. Gadolinum-enhanced T1-weighted SE images were subsequently acquired in the sagittal, axial and coronal planes [Figure 2]. There was a well-circumscribed, lobulated, capsulated, solid cystic mass lying in close proximity to the brachial plexus and vessels. No evidence of necrosis or hemorrhage was seen. The lesion was lying inferior to the head of the humerus. The superomedial surface of the lesion was indenting the subscapularis muscle along its inferolateral margin and the tendon appeared stretched out over the superior surface of the lesion. Planes between the lesion and the joint capsule of the glenohumeral joint were not well maintained. The lesion was isointense on T1-weighted sequence, hyperintense on T2-weighted sequence, heterogeneously hyperintense on fat-saturated image and showing intense homogenous enhancement on postcontrast T1W images. No perilesional edema was seen. No enlarged lymph nodes were seen.

Fine needle aspiration from the lesion done twice was inconclusive and revealed red blood cells only.

A small biopsy was required in this case for establishing a definitive diagnosis before carrying out an excision of the mass. However, as the patient had already undergone fine needle aspiration cytology (FNAC) twice and did not give consent for a third biopsy in view of a strong clinical diagnosis of a benign condition supported by the MRI scan findings, the biopsy was not performed. A decision of excision biopsy was taken after weighing the various pros and cons and explaining to the patient that in the event of the tumor being unresectable, a small biopsy only will be performed.

The patient was operated from an anterior approach to the shoulder extending up to the posterior axillary fold. The arm was kept semiabducted to avoid stretching of the axillary structures over the mass. The axillary vessels and nerves were separated carefully and the mass was dissected in the depth of the incision. The deltopectoral interval was demarcated in the upper part of the incision to reach till the coracoid process and the conjoint tendon was identified. The pectoralis muscle was not divided. Blunt dissection was performed above and below the muscle. The ascending branch of the anterior circumflex vessels was identified and cauterized. The mass was found adherent to the fibers of the subscapularis and capsule at the anteroinferior aspect of the glenoid.

The tumor was removed *en block*. The wound was closed in layers over a suction drain for 48 h. The postoperative period was uneventful. Stitches were removed on the 12^th^ day and ROM exercises were started according to his tolerance.

### Gross examination

The mass was an irregular, bosselated, grayish white, firm tissue measuring 80 mm × 55 mm × 40 mm, with few attached muscle fibers on the outer surface. On cutting open, the surface was grayish white with few circular homogenous areas.

### Microscopic examination

**Figure 3:**
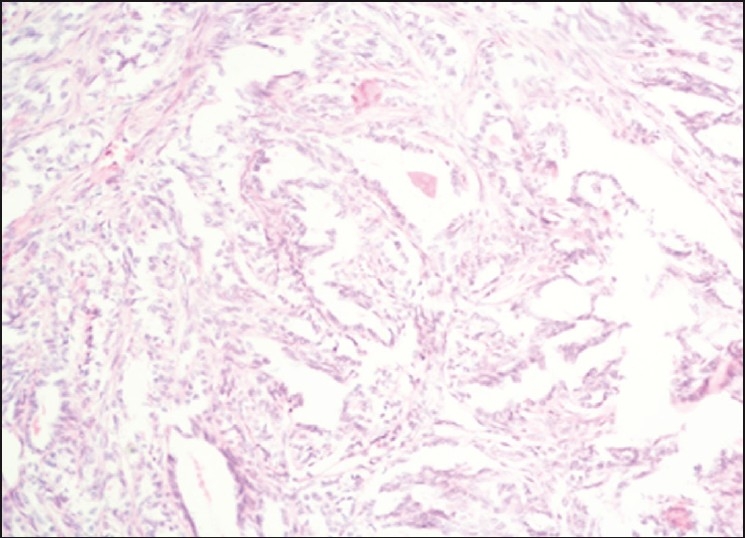
H and E stained section in ×20 magnification

**Figure 4:**
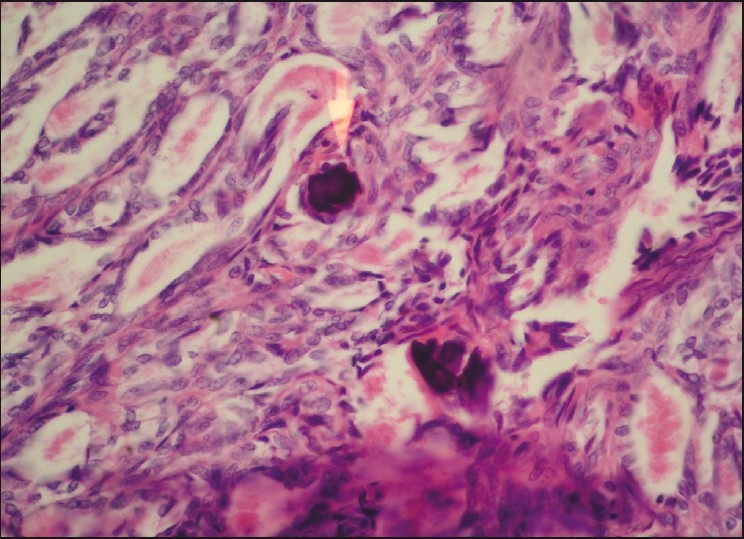
H and E stained section in ×40 magnification showing psammoma bodies in the lesion

The lesion was well circumscribed and showed a fibrous capsule on one side and fibrofatty tissue on the other side. It consisted of nodules of various sizes separated with fibrous bands. Each nodule was partly solid and partly showing glandular or cystic spaces. The latter contained papillary structures. Cuboidal cells lined the glandular spaces and papillary structures with uniform overlapping pale staining nuclei. The spaces contained eosinophilic granular material. In between the glandular spaces and in the solid areas, the cells were either polyhedral or rounded having a clear cytoplasm and rounded nuclei or oval with elongated nuclei. Many psammoma bodies or large chunks of calcification were seen at many places. Mitotic figures were rare and there was no necrosis. The histopathological diagnosis of benign NH was made in view of the above-mentioned findings typical for this tumor [Figures 3 and 4]. No further immunohistochemistry tests were done as the pathologist reported adequate evidence of this lesion being benign.

**Figure 5:**
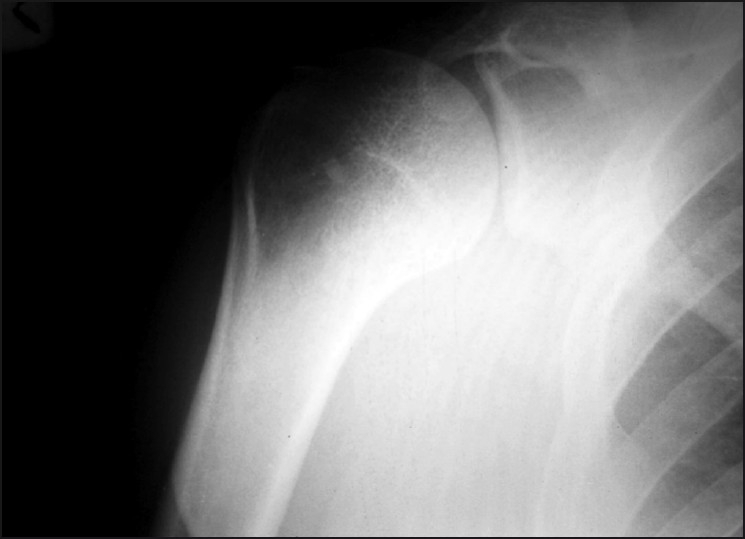
Postoperative radiograph showing absence of the calcification

**Figure 6:**
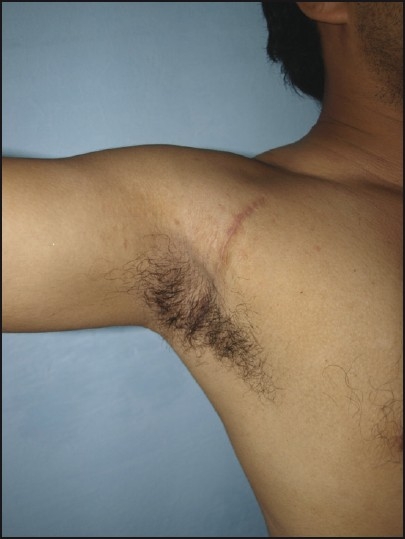
Postoperative 6-month follow-up showing the scar over the axilla

At a follow-up of 6 weeks, the patient had full ROM at the shoulder; except extreme external rotation and abduction, he was symptomatically better and there was no feeling of fullness in the axilla. At the 6-month follow-up [Figures 5 and 6], the patient had no restriction of motion and could perform activities that caused him pain earlier.

## DISCUSSION

Benign NH has many synonyms, namely solid cystic hidradenoma, clear cell myoepithelioma, eccrine sweat gland adenoma, large cell hidradenoma and eccrineacrospiroma. It affects women more commonly than men and is usually found on the scalp, face, anterior trunk and extremities. This tumor occurs at any stage of life, but is most common in the fourth decade. Skin color changes, skin thickening, serous discharge or tenderness can accompany the lesion. The lesion is superficial and hence it is diagnosed early and excised often without much investigation.[1] A deep NH of the axilla is extremely rare. It is described to be arising from the axillary tail of the breast in females.[3]

There have been reported cases of NH from various parts of the body, like eyelids, vulva, palmer aspect of the hand, ulcerated lesion on the face and axillary tail of the breast.[4–6] Malignant transformation has been described and there is a report of metastasis in the skeleton from a lesion in the foot.[2] These tumors find their description in plastic surgery or dermatological texts. They seldom grow to enormous sizes as this is dermal in origin and is frequently seen early. As far as our knowledge of articles in the English literature is concerned, this size of benign NH has not been reported. It is also the first report of this tumor in the axilla hampering the shoulder function. We kept the differential diagnoses of an enlarged lymph node, tumoral calcinosis, myositis ossificans traumatica, hydatid cyst, synovial cell sarcoma, osteochondroma or accessory breast tissue on the basis of clinical and radiographic examination. Subsequently, on MRI, a possibility of a Schwannoma, hemangioma, rhabdomyosarcoma arising from the subscapularis and cystic hygroma in adult and soft tissue fibroma were also kept and myositis, osteochondroma, tumoral calcinosis and hydatid cyst were ruled out. FNAC was hence attempted twice to establish a diagnosis before surgical removal but, in spite of it being inconclusive, core biopsy was not attempted as the patient did not give his consent.

Excision of the mass was a difficult task from an anterior approach as its location was posterior, but because vital axillary structures could be best protected from an anterior axillary approach, this was preferred. This tumor cannot be ignored as it can rarely undergo malignant transformation. In such a large tumor, of such long duration, such possibility could exist. However, there was no evidence of malignant transformation in this case as per the clinical and histopathological features. This case report is interesting in the fact that it has added NH in the differential diagnosis of an axillary mass presenting as painful restriction of motion of the shoulder joint.
